# Longitudinal Changes of Caudate-Based Resting State Functional Connectivity in Mild Traumatic Brain Injury

**DOI:** 10.3389/fneur.2018.00467

**Published:** 2018-06-19

**Authors:** Hui Xu, Xiaocui Wang, Zhen Chen, Guanghui Bai, Bo Yin, Shan Wang, Chuanzhu Sun, Shuoqiu Gan, Zhuonan Wang, Jieli Cao, Xuan Niu, Meihua Shao, Chenghui Gu, Liuxun Hu, Limei Ye, Dandong Li, Zhihan Yan, Ming Zhang, Lijun Bai

**Affiliations:** ^1^The Key Laboratory of Biomedical Information Engineering, Ministry of Education, Department of Biomedical Engineering, School of Life Science and Technology, Xi'an Jiaotong University, Xi'an, China; ^2^Department of Medical Imaging, The First Affiliated Hospital of Xi'an Jiaotong University, Xi'an, China; ^3^Department of Radiology, The Second Affiliated Hospital and Yuying Children's Hospital of Wenzhou Medical University, Wenzhou, China; ^4^Department of Neurosurgery, The Second Affiliated Hospital and Yuying Children's Hospital of Wenzhou Medical University, Wenzhou, China

**Keywords:** mild traumatic brain injury, caudate, dysfunction, longitudinal changes, a neuroimaging biomarker

## Abstract

Mild traumatic brain injury (mild TBI) is associated with dysfunctional brain network and accumulating evidence is pointing to the caudate as a vulnerable hub region. However, little is known about the longitudinal changes in the caudate-based resting-state functional connectivity following mild TBI. In the current study, 50 patients with mild TBI received resting-state functional magnetic resonance imaging as well as neuropsychological assessments within 7 days post-injury (acute phase) and 1 month later (subacute phase). Thirty-six age- and gender- matched healthy controls underwent the same protocol. The caudate was segmented into the dorsal and ventral sub-regions based on their related functionally distinct neural circuits and separate functional connectivity was investigated. Results indicated that patients with mild TBI at acute phase exhibited reduced left dorsal caudate-based functional connectivity with ventral lateral prefrontal cortex, dorsal anterior cingulate cortex, and inferior parietal lobule, which mainly distributed in the cognitive control network, and reduced right ventral caudate-based functional connectivity with the dorsal lateral prefrontal cortex, dorsal anterior cingulate cortex (dACC), and bilateral ventral anterior cingulate cortex (vACC), which mainly distributed in the executive network and emotional processing network. Furthermore, patients with mild TBI presented the reduced functional connectivity between the left dorsal caudate and the ventral lateral prefrontal cortex (vlPFC) compared with healthy controls at acute phase while this difference became no significance and return to the normal level following 1 month post-injury subacute phase. Similarly, the functional connectivity between the right ventral caudate and anterior cingulate cortex (both dorsal and ventral part) showed the reduced strength in patients compared with healthy controls only at the acute phase but presented no significant difference at subacute phase following mild TBI. Along the same line, patients with mild TBI presented the impaired performance on the information processing speed and more complaints on the pain impact index at acute phase compared with healthy controls but showed no significant difference at the follow-up 1 month post-injury subacute phase. The longitudinal changes of caudate-based dysfunction connectivity could serve as a neuroimaging biomarker following patients with mild TBI, with the evidence that the abnormal caudate-based functional connectivity at acute phase have returned to the normal level accompanying with the recovery of the neuropsychological syndromes following patients with mild TBI at subacute phase.

## Introduction

Mild traumatic brain injury (mild TBI) is a vital public health care problem (1), accounting for almost 80% of traumatic brain injuries (2). A considerable number of mild TBI with negative conventional clinical neuroimaging findings develop various neuropsychological impairments mostly in the cognitive controls (3, 4), attention (5), executive functions (6), emotion (7), working memory (8), and prospective memory (9).

These cognitive and emotion deficits have been suggested to be caused by damages to brain functional connectivity (10–14), particularly due to disconnections of vital network hubs (15, 16) following mild TBI. However, accumulating evidence is pointing to the caudate as a most vulnerable hub region following mild TBI. One recent DTI study using graph theory to characterize brain connectivity demonstrates that betweenness centrality and eigenvector centrality are reduced within network hubs, particularly evident within hub region such as the caudate (15). Moreover, evidence from structural volume analysis and DTI analysis indicates that the structural integrity of cortical-subcortical circuits can account for executive impairments following mild TBI. Greater local atrophy volume within the caudate is related with severe impairment in individual execution function (17), and the decreased FA of fiber tract between caudate and superior frontal gyrus is associated with increased switching errors (18). In addition, disruption of caudate activation is also associated with a worse performance in working memory when chronic blast TBI receives a Sternberg Item Recognition Task. This study also indicates that the caudate is a specific vulnerability to blast injury, and may serve as a biomarker for blast TBI (19). Nonetheless, involvement of the caudate-based functional connectivity in the cognitive deficits following mild TBI has not been investigated.

The caudate plays a critical role in various cognitive functions, and can be segmented into dorsal and ventral sub-regions based on their related functionally distinct neural circuits (20, 21). The dorsal caudate, a key component of the dorsal striatum, is anatomically and functionally connected with high level cortical regions such as the dorsal or ventral lateral prefrontal cortex, anterior cingulate cortex, and inferior parietal lobule, and their functional pathways contributed to cognitive controls (22, 23). Whereas, the ventral caudate, a component of the ventral striatum, is closely linked to the dorsolateral prefrontal cortex, inferior frontal gyrus, rostral anterior cingulate, and posterior cingulate cortex, and their different neural circuits are implicated in executive function and emotional processing respectively (24, 25). However, the functional connectivity based on the differential sub-sections of the caudate has received less attention. Furthermore, there have been only a few longitudinal imaging studies performed to find the longitudinal changes and recovery among functional connectivity between early and later phases of mild TBI compared to the course of the healthy controls, but with mixed findings (13, 26–28), mainly due to the whole brain functional connectivity lacking precision brain hub regions concerned.

Building on previous findings and limitations, a prospective and longitude cohort study based on the caudate-based functional connectivity was performed. Firstly, we investigated whether caudate-based functional connectivity was disrupted in patients with mild TBI compared with healthy controls at acute phase. Secondly, we evaluated the longitudinal changes of the altered caudate-based functional connectivity in patients with mild TBI from acute phase to subacute phase, and further examined whether the longitudinal changes normalized as a function of recovery. Finally, we examined how the longitudinal changes of the altered caudate-based functional connectivity related to the performance on neuropsychological measures in patients with mild TBI.

## Methods

### Participants

A total of fifty patients with mild TBI (30 male, mean age of 37.2 ± 12.6 years, education level of 10.3 ± 4.2 years) and 35 age-, education-matched healthy controls (HC, 14 male, mean age of 35.3 ± 10.9 years, education level of 11.6 ± 5.5 years) participated in the study. HC were recruited through public advertising.

All consecutively patients with non-contrast head CT due to acute head trauma enrolling from the local emergency department (ED) formed the initial population. Screening for mild TBI was based on the World Health Organization's Collaborating Centre for Neurotrauma Task Force (29). The inclusion criteria included: (i) Glasgow Coma Score of 13–15; (ii) one or more of the following: loss of consciousness (if present) <30 min, post-traumatic amnesia (if present) <24 h, and/or other transient neurological abnormalities such as focal signs, seizure, and intracranial lesion not requiring surgery. Mild TBI participants were excluded following the criteria: history of neurological disease, long-standing psychiatric condition, head injury, or a history of substance or alcohol abuse, intubation and/or presence of a skull fracture and administration of sedatives on arrival in the emergency department, spinal cord injury, the manifestation of mild TBI due to medications by other injuries (e.g., systemic injuries, facial injuries, or intubation) or other problems (e.g., psychological trauma, language barrier, or coexisting medical conditions), or caused by penetrating craniocerebral injury. In addition, healthy control subjects with no history of neurological or psychiatric disorder were also recruited. Participants were all right-handed according to the Edinburgh Handedness Inventory (30). All the subjects gave written, informed consent in person approved by a local institutional review board and conducted in accordance with the Declaration of Helsinki.

MRI scanning for patients with mild TBI were initially evaluated within 7 days post-injury (acute phase) and follow-up at 1 month after injury (subacute phase). Neuropsychological tests were performed within 48 h of MR imaging. Within the same time interval, HC completed the identical assessments as patients with mild TBI.

### Neuropsychological tests

Comprehensive neuropsychological tests were assessed: (i) Trail-Making Test Part A (31) to examine cognitive information processing speed; (ii) Forward Digit Span and Backward Digit Span of the Wechsler Adult Intelligence Scale WAIS-III (32) to assess working memory; (iii) Digit Symbol Coding (DSC) task (33) to assess memory and information processing speed. Self-reported symptomatology included: the Rivermead Post-Concussion Symptoms Questionnaire (RPQ) (34), the Insomnia Severity Index (ISI) (35), the Short-Form Headache Impact Test (36).

### Image acquisition

A non-contrast CT scan was performed on all consecutive patients following acute head injury with a 64-row CT scanner (GE, Lightspeed VCT). The MRI scans were acquired with the use of 3.0T MRI scanner (GE 750) with a total scan time of 32:03 min. A custom-built head holder was used to prevent head movements. All participants were instructed to remain in a relaxed state without engaging in cognitive or motor activity and to keep their eyes closed. Alertness during the scan was confirmed immediately afterward. In this study, the MRI protocol involved the high-resolution T1-weighted 3D MPRAGE sequence [echo time (TE) = 3.17 ms, repetition time (TR) = 8.15 ms, flip angle = 9°, slice thickness = 1 mm, field of view (FOV) = 256 × 256 mm, matrix size = 256 × 256, acquisition time = 4:30 min], a single-shot, gradient-recalled echo planar imaging (EPI) sequence with a total of 180 volumes of 54 slices covering the whole brain (TR = 2,500 ms, TE = 30 ms, slice thickness = 3 mm, flip angle = 90°, FOV = 216 mm × 216 mm, matrix size = 64 × 64, voxel size = 3 mm × 3 mm × 3 mm, acquisition time = 7:30 min), and diffusion weighted imaging (TR = 7,300 ms, TE = 99 ms, flip angle = 90°, thickness = 3 mm, slices = 50, FOV = 256 mm × 256 mm, matrix size = 128 × 128, two averages, voxel size = 2 mm × 2 mm × 3 mm). DTI scan (b = 1,000 s/ mm^2^) were acquired with 30 diffusion gradient orientations and the b = 0 repeated two times (acquisition time = 9:28 min) and 3D ASL including M0 image and perfusion different image with the paramaters (TR = 5,046 ms, TE = 11 ms, slice thickness = 3 mm, field of view (FOV) = 24 × 24 mm, labeling time = 1.5 s, post-labeling delay = 2,000 ms, acquisition time = 4:53 min). The presence of focal lesions and cerebral microbleeds was independently determined by experienced clinical neuroradiologists (with 9 and 10 years' experience) who assessed multiple modalities of neuroimaging data acquired at baseline (T1-flair, T2-flair, T2, susceptibility weighted imaging, total acquisition time = 5:42 min).

### MRI data preprocessing

For each participant, resting-state fMRI images were preprocessed according to the following steps. The first 10 functional scans were discarded to eliminate transients and account for T1 relaxation effects. The remaining functional images were preprocessed using standard protocols in FSL v 5.0 (FMRIB's Software Library, www.fmrib.ox.ac.uk/fsl) and included the following steps: (1) slice timing was performed to compensate for acquisition delays across slices; (2) motion artifacts of timing corrected images were estimated and corrected by realigning all functional images to the middle image; (3) linear-spatial normalization to MNI space by using unified segmentation of the high-resolution T1-weighted anatomical image (3D MPRAGE); (4) voxel re-sampling to 2 × 2 × 2 mm resolution; (5) spatial smoothing with a 6 mm full-width-half-maximum (FWHM) Gaussian kernel; (6) band-pass temporal filtering (0.01–0.1 Hz): removal of very low (<0.01 Hz) and high frequencies band (>0.1 Hz) with a finite impulse response (FIR) filter, which was reported to be of physiological importance (37, 38); (7) nuisance signal regression: the time series of nine nuisance signals were regressed out in the functional connectivity analyses, including six head motion parameters and three averaged signals representing white matter (WM), cerebrospinal fluid (CSF), and global signal, and the first temporal derivatives of aforementioned parameters. The six head motion parameters were obtained from the motion correction preprocessing step, expressed as absolute differences from the middle time point in each of the three translational and rotational directions. In order to extract the nuisance covariate time series for the WM, CSF, and global signal, each individual's high-resolution T1-weighted anatomical image was segmented by using FSL's FAST segmentation program. The resulting segmented WM and CSF images were then threshold to ensure 80% tissue type probability. The mean time series was calculated by averaging across all voxels within the threshold masks among each individual's time series.

### Functional connectivity: caudate-based seeds

**Figure 1:**
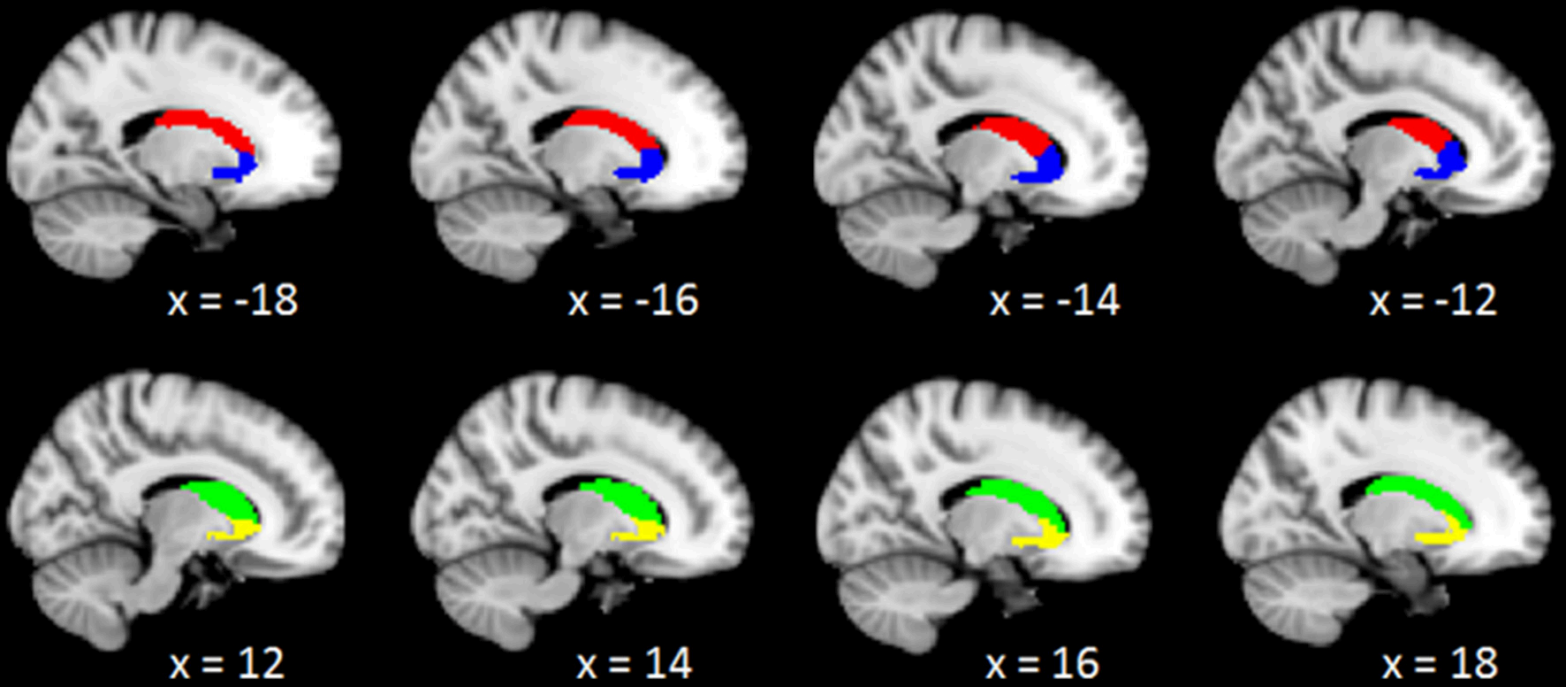
Representation of the 4 caudate regions of interest. The above panel shows the projection of the 2 left caudate regions, left dorsal caudate (red), and left ventral caudate (blue), onto sagittal brain views for x = −18, −16, −14, −12; The below panel shows the projection of the 2 right caudate regions, right dorsal caudate (green), and right ventral caudate (yellow), onto sagittal brain views for x = 12, 14, 16, 18, respectively.

Neuroimaging studies indicated the caudate consisted of three parts: head, body, and tail (39). Specific subdivisions of the caudate are known to be functionally connected to various cortical networks (40–42). The disruption to cortico-caudate loops produces executive dysfunction in patients with TBI. A recent study finds reduced functional connectivity was particularly observed between caudate subdivision and the cingulate cortex (43). In order to precisely investigate the longitudinal changes of caudate-based functional connectivity in patients with mild TBI, we used the bilateral caudate seeds, which were divided into dorsal and ventral sub-regions, according to the Brainnetome atlas in the standard MNI template space (44). These caudate seeds were shown in the Figure 1, the left dorsal caudate centered at the Montreal Neurological Institute coordinates [−14 2 16], the left ventral caudate centered at [−12 14 0], the right dorsal caudate centered at [14 5 14], the right ventral caudate centered at [15 14 −2] (see Table 1 for seed coordinates).

**Table 1:** Coordinates for caudate regions of interest.

**Seed**	**MNI coordinates (mm)**		
	**x**	**y**	**z**
Left dorsal caudate	−14	2	16
Right dorsal caudate	14	5	14
Left ventral caudate	−12	14	0
Right ventral caudate	15	14	−2

*MNI, Montreal Neurological Institute*.

### Statistical analysis of functional connectivity

Resting-state functional connectivity was performed by using custom MATLAB scripts. For each caudate seed, time series was the averaged time course of all voxels within each seed region for each participant. Then Pearson's correlation coefficients between each caudate seed's time series and the time series of every voxel across the whole brain were calculated for each individual. These resulting correlation coefficients were later transformed to Z-scores using Fisher's transformation. The Fishers-Z-scores of correlation coefficients were expressed as functional connectivity strength.

Group level two-sample *t*-tests were firstly performed to compare functional connectivity based on each caudate seed between mild TBI and HC population at acute phase. Functional connectivity statistical maps were defined using non-parametric permutation testing, thresholded using the threshold-free cluster enhancement (TFCE) method and corrected for multiple comparisons with a family-wise error (FWE) rate of *p* < 0.05. The 2 × 2 [Group (mild TBI, HC) × Time (acute phase, subacute phase)] mixed measures ANOVAs were then analyzed to examine whether caudate-based functional connectivity longitudinal changed to normalization as a function of recovery. The analyses were restricted to the functional connectivity as they exhibited significant differences between mild TBI and HC at acute phase.

### Statistical analysis of behavior

Statistical analyses of neuropsychological assessments were performed in SPSS v23.0 (Statistical Package for the Social Sciences, IBM, New York, USA). Group level two-sample *t*-tests were performed to investigate whether patients with mild TBI would exhibit worse performance at acute phase relative to healthy controls. Then the 2 × 2 [Group (mild TBI, HC) × Time (acute phase, subacute phase)] mixed measures ANOVAs were conducted to examine changes in self-reported symptomatology and cognitive function as a function of recovery in patients with mild TBI.

## Results

### Participant characteristics

All demographic and clinical characteristics for patients with mild TBI and HC were presented in the Table 2. At acute phase in the emergency department, all patients with mild TBI had an initial GCS of 15. Mechanisms of injury included: 62% motor vehicle accidents (MVA), 24% assault, and 14% falls. The presence of focal lesions and cerebral microbleeds was independently determined by experienced clinical neuroradiologists (with 9 and 10 years' experience) who assessed multiple modalities of neuroimaging data acquired at acute phase (T1, T2, FLAIR, susceptibility weighted imaging). Any disagreement between these two observers was resolved by consensus. None of patients were with visible contusion lesions using conventional neuroimaging techniques or exhibited cerebral micro-bleeds on SWI. There were no differences in age [*t*_(__84)_ = 0.721, *p* > 0.05], education level [*t*_(84)_ = −1.202, *p* > 0.05], or gender [$\chi_{\left(1\right)}^{2}$ = 3.733, *p* > 0.05] between mild TBI and HC.

**Table 2:** Summary of demographic characteristics, neuropsychological test scores between HC and mild TBI participants.

**Characteristic**	**HC (*n* = 36)**		**Mild TBI (*n* = 50)**	
Age (years)	35 ± 10		37 ± 12	
Gender				
Male	14 (39%)		30 (60%)	
Female	22 (61%)		20 (40%)	
Handedness				
Right	36		50	
Left	0		0	
Education (years)	11.6 ± 5.5		10.3 ± 4.2	
**Scan timepoint**	**Acute phase**	**Subacute phase**	**Acute phase**	**Subacute phase**
**NEUROPSYCHOLOGICAL TEST**				
Processing Speed				
TMT A	46.2 ± 33.3	37.8 ± 23.4	65.4 ± 45.8	55.9 ± 46.1
DCS	46.6 ± 16.1	48.5 ± 15.7	33.5 ± 15.7	40.1 ± 17.1
Working Memory				
FDS	8.3 ± 1.5	8.8 ± 1.6	7.6 ± 1.6	8.1 ± 1.5
BDS	4.4 ± 1.8	4.5 ± 1.6	3.6 ± 1.5	4.1 ± 1.8
Symptom Severity				
RPQ	2.5 ± 2.5	1.5 ± 1.8	9.6 ± 6.3	6.3 ± 5.4
ISI	1.9 ± 3.2	1.7 ± 2.6	7.2 ± 5.9	5.3 ± 5.6
HIT-6	37.2 ± 3.6	36.3 ± 2.0	47.8 ± 8.8	40.9 ± 7.1

*Values given as mean ± standard deviation. TMT-A, Trail-Making Test Part A; DSC, Digit Symbol Coding Task; FDS, Forward Digit Span Task; BDS, Backward Digit Span Task; RPQ, Rivermead Post-Concussion Symptoms Questionnaire; ISI, Insomnia Severity Index; HIT-6, Short-Form Headache Impact Test*.

**Table 3:** Clusters demonstrating differences in caudate-based functional connectivity between mild TBI and HC participants at acute phase.

**Seed**	**Region**	**Side (L/R)**	**Size (voxels)**	**Peak MNI coordinates (mm)**		
				**x**	**y**	**z**
**MILD TBI < HC PARTICIPANTS AT ACUTE PHASE**						
Left dorsal caudate	vlPFC	R	73	38	16	−12
	dACC	R	24	4	32	−8
	IPL	R	39	67	−34	22
Right ventral caudate	dlPFC	L	84	−48	40	26
	dACC	L	40	−12	29	30
	vACC	L	125	−2	1	30
		R	97	8	−8	38

*vlPFC, ventral lateral prefrontal cortex; dACC, dorsal anterior cingulate; IPL, inferior parietal lobule; dlPFC, dorsal lateral prefrontal cortex; vACC, ventral anterior cingulate; L/R, left/right hemisphere; BA, The Brodmann area; MNI, Montreal Neurological Institute*.

### Neuropsychological measures

Patients with mild TBI presented significantly worse performance on scales of the RPQ, ISI, short-form headache impact test (HIT-6) at acute phase, compared with healthy controls (*p* < 0.005). There were also significant differences in performance on information processing speed reflected by the DSC task (*p* < 0.005).

**Figure 2:**
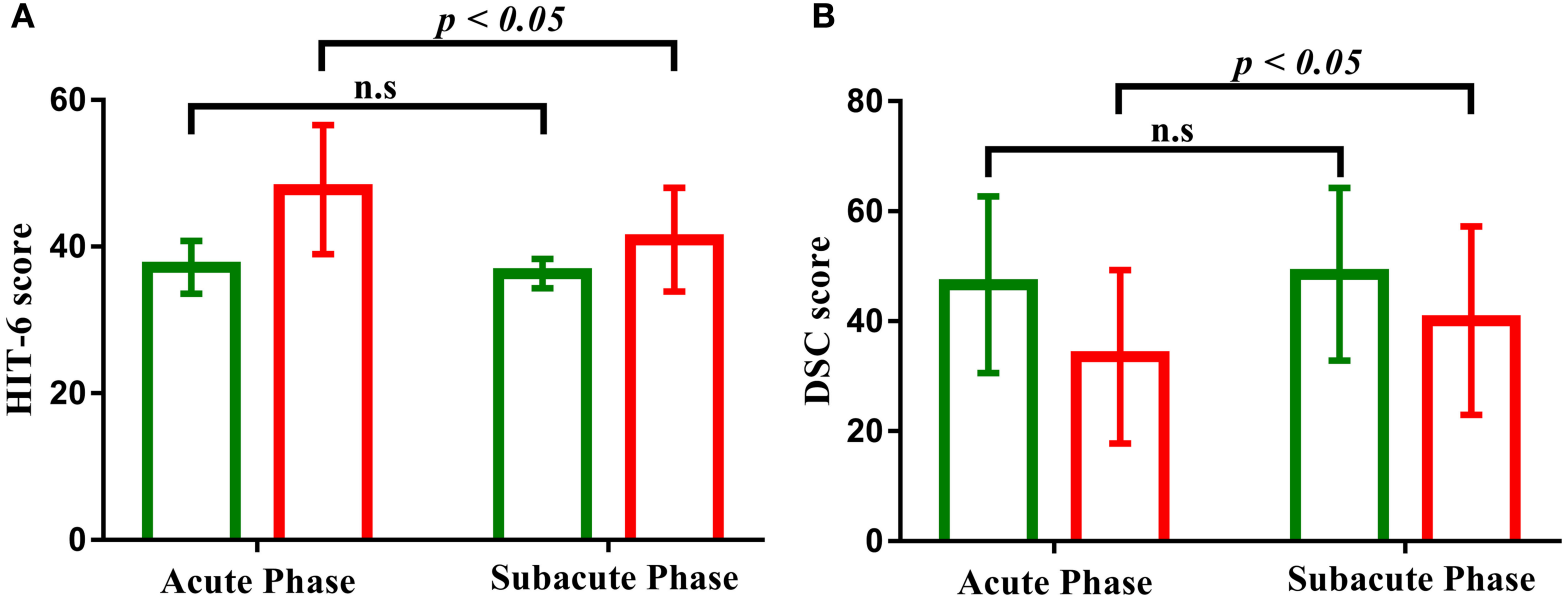
**(A)** Bar graph shows the decreased performance of clinical symptom (HIT-6) scales as a function of time (acute phase to subacute phase) with patients with mild TBI (red) but not healthy controls (green). **(B)** Bar graph shows the increased performance of cognitive function (DSC) scales as a function of time (acute phase to subacute phase) with patients with mild TBI (red) but not healthy controls (green). “n.s” marks *p* > 0.05, and error bar represents standard deviations of the mean.

Longitudinal analyses were then conducted to examine changes in self-reported symptomatology and cognitive function as a function of recovery. The main effects of time were significant in the HIT-6 and DSC scores (all *p* < 0.001). The Group × Time interaction was also significant for self-reported symptomatology (HIT-6) [*F*_(1, 84)_ = 18.615, *p* < 0.001, Figure 2A], and information processing speed (DSC) [*F*_(1, 84)_ = 4.804, *p* < 0.05, Figure 2B], with simple effects testing suggested that the level of self-reported symptomatology decreased and information processing speed increased as a function of time (acute phase to subacute phase) within patients with mild TBI (all *p* < 0.05) but not healthy controls (all *p* > 0.05). Both the measure of the HIT-6 and DSC showed significantly abnormal in patients compared to healthy controls at acute phase (all *p* < 0.05) but not follow-up subacute phase (all *p* > 0.05). Other measures presented no significant of time, group or interaction effect.

### Functional connectivity analyses—group comparison at acute phase

#### Dorsal caudate seeds based functional connectivity

**Figure 3:**
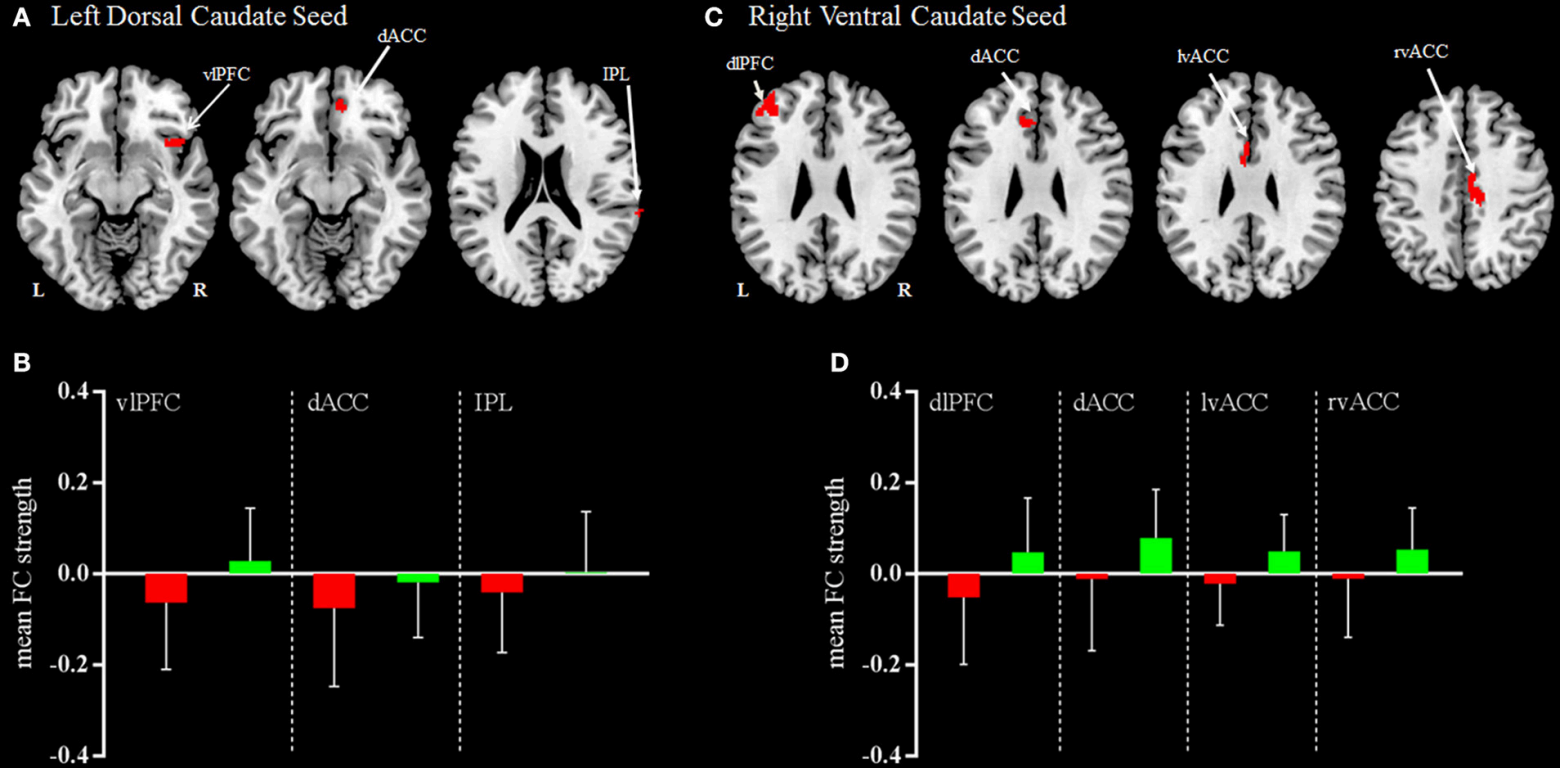
Regions demonstrating group differences in mean FC (functional connectivity) strength for the left dorsal caudate seed **(A)** and right ventral caudate seed **(C)** at acute phase. Red coloring indicates regions where the functional connectivity strength was significantly decreased for patients with mild TBI, compared with HC. The bar charts display mean functional connectivity strength between selected significant regions within the left dorsal caudate seed **(B)** and right ventral caudate seed **(D)** above for mild TBI (red) and HC (green). Error bars illustrate standard deviations of the mean. Coordinates for slice locations are presented according to the MNI atlas, and cluster volumes are presented in Table 3. L,Left; R,Right.

Compared with HC, patients with mild TBI demonstrated significantly decreased functional connectivity between the left dorsal caudate and ventral lateral prefrontal cortex (vlPFC), dorsal anterior cingulate cortex (dACC), and inferior parietal lobule (IPL), while no increased functional connectivity (Figures 3A,B). In addition, no significant differences were observed in the right dorsal caudate-based functional connectivity in patient with mild TBI relative to HC.

#### Ventral caudate seeds based functional connectivity

Compared with HC, patients with mild TBI demonstrated significantly decreased functional connectivity between the right ventral caudate and the dorsal lateral prefrontal cortex (dlPFC), dACC, and bilateral ventral anterior cingulate cortex (vACC), while no increased functional connectivity (Figures 3C,D). In addition, no significant differences were observed in the left ventral caudate-based functional connectivity in patients with mild TBI compared to HC.

### Functional connectivity longitudinal changes as function of recovery within mild TBI

The 2 × 2 mixed measures analysis were performed to determine whether there was a significant recovery in the functional connectivity following patients with mild TBI at subacute phase.

**Figure 4:**
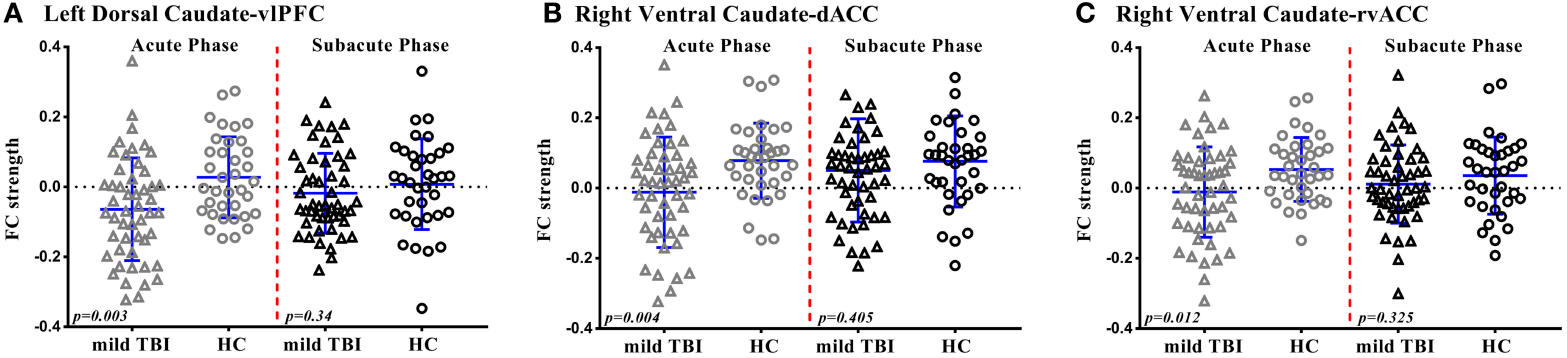
Scatterplots of Functional Connectivity (FC) strength of significant regions are displayed for mild TBI (open triangles) and HC (open circles) at acute phase and subacute phase. Each symbol represents a single individual's FC strength. The I bars indicate the means and standard deviation. The *p*-values were obtained from group mean difference. **(A)** Left dorsal caudate-vlPFC functional connectivity. **(B)** Right ventral caudate-dACC functional connectivity. **(C)** Right ventral caudate-rvACC functional connectivity.

For the left dorsal caudate, the decreased functional connectivity with the vlPFC was significantly recovered to HC baseline level in patients after 1 month post-injury (*p* > 0.05, Figure 4A), whereas the left dorsal caudate-dACC or IPL functional connectivity was not.

For the right ventral caudate, the decreased functional connectivity with the dACC and rvACC was significantly recovered to HC baseline level in patients at subacute phase (*p* > 0.05, Figures 4B,C), whereas the right ventral caudate-dlPFC or lvACC functional connectivity was not.

### Relationship between functional connectivity and neuropsychological measures over phase

No significant linear correlations were observed between the caudate-based functional connectivity and neuropsychological measures in patients with mild TBI at acute phase (*p* > 0.05) or subacute phase (*p* > 0.05). Nonetheless, the abnormal changes in functional connectivity have returned to the normal level accompanying with the recovery of the neuropsychological syndromes (HIT-6, DSC) in patients with mild TBI.

## Discussion

To the best of our knowledge, this is the first study to investigate the longitudinal changes in the caudate-based resting-state functional connectivity from acute phase to subacute phase in a relatively large sample of 50 patients with mild TBI and 36 healthy controls. Results found longitudinal changes of caudate-based functional connectivity which could serve as a neuroimaging biomarker in patients with mild TBI. First, we observed that patients with mild TBI exhibited reduced left dorsal caudate-based functional connectivity with vlPFC, dACC, and IPL, which mainly distributed in the cognitive control network, and reduced right ventral caudate-based functional connectivity with dlPFC, dACC, and bilateral vACC, which mainly distributed in the executive network and emotional processing network. Furthermore, we demonstrated the altered left dorsal caudate-based functional connectivity with vlPFC showed longitudinal changes and exhibited a significant recovery, similarly the right ventral caudate-based functional connectivity with the dACC and rvACC. Additionally, we detected that the abnormal changes in functional connectivity have returned to the normal level accompanying with the recovery of the neuropsychological syndromes (HIT-6, DSC) in patients with mild TBI.

### Caudate-based functional connectivity differences of mild TBI in comparison with HC at acute phase

Neuroimaging studies suggest the caudate consists of three parts: head, body, and tail (39). Recent studies propose to divide the caudate into dorsal and ventral portions because of different caudate parts related to distinct cortical areas (40–42). By systematically exploring striatal organization with functional connectivity, the dorsal caudate is primarily connected with the dorsolateral prefrontal cortex, ventral lateral prefrontal cortex, anterior cingulate, posterior cingulate, and inferior parietal lobule, while the ventral caudate functionally correlated with the dorsolateral prefrontal cortex, inferior frontal gyrus, rostral anterior cingulate, and posterior cingulate cortex (22, 45, 46).

More neuroimaging studies prove that the dorsal caudate is associated with cognitive control and information processing speed, connecting with the ventral prefrontal cortex, dorsal prefrontal cortex, dorsal anterior cingulate, and IPL (21, 22, 42, 47). Consistent with previous findings, our study indicated that patients with mild TBI demonstrated significantly decreased functional connectivity within the left dorsal caudate-based cognitive control network than HC. Meanwhile, patients with mild TBI at acute phase showed significantly worse performance in the information processing speed and cognitive control. These findings implied that the decreased functional connectivity among cognitive control network resulted in worse performance of its related neuropsychological assessment in patients with mild TBI.

In contrast, the ventral caudate is examined to be more functionally connected to the ventral anterior cingulate, involving in the affective and emotional processing (20, 48), and further connected to the dorsal prefrontal cortex and dorsal anterior cingulate, involving in the executive function (22, 49). Similarly, our findings indicated thatpatients with mild TBI showed significantly the decreased right ventral caudate-based functional connectivity than HC in the dlPFC, dACC, and bilateral vACC. Further, patients with mild TBI at acute phase showed significantly worse self-reported symptomatology of emotion. These findings implied that the decreased functional connectivity among emotional processing network resulted in worse performance of its related self-reported symptomatology assessment in patients with mild TBI.

### Changes of caudate-based functional connectivity in mild TBI at subacute phase

The group × time mixed measures analysis observed a functional connectivity recovery in patients with mild TBI after 1 month post-injury. Nevertheless, only part of caudate-based networks with the reduced functional connectivity at acute phase was observed a significant recovery. However, the reduced functional connectivity had not returned to the normal level after 1 month post-injury. A potential mechanism (50) for the changes explained that a transfer from global to more local brain communication, especially for hub-regions network probably happened in mild TBI at the subacute phase in order to alleviate the hub overload of the highest nodes in the hierarchy and begin to reroute information traffic to nodes at a lower order. The mechanism could explain the caudate was a specific vulnerability hub to brain injury, and part of caudate-based networks showed a significant recovery.

Nevertheless, our findings were in line with prior longitudinal studies that discovered a recovery in whole-brain functional connectivity after 6 months, which found functional connectivity between right superior frontal gyrus and left caudate recovered between 3 and 6 months after injury (27), while other studies failed to detect prospective changes in functional connectivity during a 4-month (13), and a 6-month period (51). However, the reasons for this variability could be the differences between seed-based functional connectivity and whole-brain functional connectivity analysis and the time difference after injury.

### Relationship between functional connectivity and cognition over phase

The relationship between altered pattern of functional connectivity and deficit cognition performance, especially in attention, executive function, and working memory has been supported in patients with mild TBI (10, 52–54), however few studies investigated the correlation between changes in functional connectivity and changes in cognitive behavioral measures over a long time period (55, 56).

The current study examined longitudinal changes of caudate-based functional connectivity in patients with mild TBI, which may be connected with cognitive and related symptomatology improvement. Unexpectedly, no significant linear correlations between changes in caudate-based functional connectivity and changes in neuropsychological measures scores were observed. Nonetheless, the abnormal changes in caudate-based functional connectivity have returned to the normal level accompanying with the recovery of the neuropsychological syndromes in patients with mild TBI. We suppose this might be the consequence of the difference of recovery process about functional connectivity and cognitive function as well as related symptomatology in patients with mild TBI. Another plausible explanation would be the subjectivity of neuropsychological measures could not reflect cognitive function and symptomatology, lacking precise measuring. However, the longitudinal changes of caudate-based functional connectivity could underline the improvement of cognitive performance and related symptomatology in patients with mild TBI to some extent.

## Conclusions and additional considerations

The present study presented the longitudinal changes evidence of caudate-based functional connectivity in a homogenous sample of patients with mild TBI. These data demonstrated that patients with mild TBI were associated with a recovery of caudate-based dysfunction connectivity, accompanying with the recovery of the neuropsychological syndromes. The findings of longitudinal changes of caudate-based functional connectivity in patients with mild TBI indicated a possible reorganization of brain networks after injury. Taken into all evidence in this study, it could be suggested that the longitudinal changes of caudate-based dysfunction connectivity could serve as a neuroimaging biomarker following patients with mild TBI.

The results of this study must be tempered by some limitations. One limitation was that the differences of phases after the trauma, and changes can be observed in minutes to months after the trauma. In our study, we only investigated changes of caudate- based functional connectivity from 7 days post-injury to 1 month post-injury. However, whether the observed changes can extend beyond the 1 month time point that we have chosen, is still under debate. Since mild TBI renders large scale brain network, the further study will focus on the brain connectome scale changes. Another potential limitation was that in our study, only rest-stating fMRI data was investigated, and the results could not disprove the possibility of structural changes in the caudate, which can be measured by diffusion-tensor imaging. Future studies must explore the relationship between structural and functional network deficits as well as their clinical implication. Finally, the different parcellation way of the caudate may exert an influence on the final seed-based functional connectivity maps. However the fined division of the caudate also need a relative sample of healthy controls to pursuit this purpose. Therefore, our further study will focus on the fined parcellation of caudate sub-regions.

## Ethics statement

The research procedures were approved by the Ethical Committee of The Second Affiliated Hospital of Wenzhou Medical University and conducted in accordance with the Declaration of Helsinki.

## Author contributions

HX performed the experiment, analyzed image data, performed statistical results, and drafted the manuscript. XW, ZC, GB, BY, SW, CS, SG, ZW, JC, XN, MS, CG, LH, LY, DL, and ZY performed the experiment and collected the data. MZ and LB designed the study and gave critical comments on the manuscript.

### Conflict of interest statement

The authors declare that the research was conducted in the absence of any commercial or financial relationships that could be construed as a potential conflict of interest.
